# Therapeutic potential of targeting Tfr/Tfh cell balance by low-dose-IL-2 in active SLE: a post hoc analysis from a double-blind RCT study

**DOI:** 10.1186/s13075-021-02535-6

**Published:** 2021-06-11

**Authors:** Miao Miao, Xian Xiao, Jiayi Tian, Yunzhi Zhufeng, Ruiling Feng, Ruijun Zhang, Jiali Chen, Xiaoying Zhang, Bo Huang, Yuebo Jin, Xiaolin Sun, Jing He, Zhanguo Li

**Affiliations:** 1 Department of Rheumatology & Immunology, Peking University People’s Hospital, Beijing, 100044 China; 2 Beijing Key Laboratory for Rheumatism Mechanism and Immune Diagnosis (BZ0135), Beijing, China

**Keywords:** Systemic lupus erythematosus, T follicular helper cell, T follicular regulatory cell, Low-dose interleukin-2

## Abstract

**Objective:**

To investigate the regulation of T follicular regulatory (Tfr) and T follicular (Tfh) cell subtypes by low-dose IL-2 in systemic lupus erythematosus (SLE) in a randomized, double-blind, placebo-controlled clinical trial.

**Methods:**

A post hoc analysis was performed in a randomized cohort of SLE patients (n=60) receiving low-dose IL-2 therapy (n=30) or placebo (n=30), along with the standard of care treatment. The primary endpoint was the attainment of SLE responder index-4 (SRI-4) at week 12 in the trial. Twenty-three healthy controls were enrolled for T cell subset detection at the same time as the trial. The t-stochastic neighbor embedding (tSNE) analysis of CD4 T subsets based on immune cells flow cytometry markers was performed to distinguish Tfh, Tfh1, Tfh2, Tfh17, and Tfr cell subsets.

**Results:**

Compared with HC, the frequency of Tfr (CXCR5^+^PD-1^low^ Treg and CXCR5^+^PD-1^high^ Treg) cells was significantly reduced, while the pro-inflammatory Tfh cells were increased in patients with SLE. The imbalanced Tfh cell was associated with several pathogenic factors (anti-dsDNA antibodies (r=0.309, P=0.027) and serum IL-17 (r=0.328, P=0.021)) and SLE Disease Activity Index (SLEDAI) score (r=0.273, P=0.052). Decreased CXCR5^+^PD-1^low^ Treg/Tfh and CXCR5^+^PD-1^low^ Treg/Tfh17 were both associated with increased immunoglobulin M (IgM) (r=−0.448, P=0.002 and r=−0.336, P=0.024, respectively). Efficacy of low-dose IL-2 therapy was associated with a restored Tfr/Tfh cell balance.

**Conclusion:**

These data support the hypothesis that promotion of Tfr is associated with decreased disease activities and that low-dose IL-2 therapy can recover Tfr/Tfh immune balance.

**Trial registration number:**

ClinicalTrials.gov Registries (NCT02465580).

**Supplementary Information:**

The online version contains supplementary material available at 10.1186/s13075-021-02535-6.

## Key messages

1. Deregulation between Tfr and Tfh subsets associated with severity of SLE.
2. Low-dose IL-2 therapy was efficient in patients with SLE.
3. Low-dose IL-2 therapy elevates the Tfr/Tfh ratio, which might be a novel concept to design the therapeutic regimen.

## Introduction

Systemic lupus erythematosus (SLE) is characterized by the breakdown of immune tolerance leading to auto-reactive immune responses and consequently, tissue and organ damages. Over the past decades, extensive studies on regulatory T (Treg) cells have revealed that these cells can maintain tolerance and regulate immune responses [1, 2], while T follicular helper cells (Tfh) play an important role in the production of autoantibodies and pro-inflammatory cytokines in SLE [3–5]. Moreover, the imbalance of the immune response between pro-inflammatory and anti-inflammatory cells is central in SLE pathogenesis.

T follicular regulatory (Tfr) cells share features with Tfh and conventional Treg cells and can inhibit Tfh cells and germinal center (GC) responses with a significant impact in humoral immunity [6–8]. Previous studies have suggested that Tfr cells can be identified as CXCR5^+^PD-1^low^ Treg and CXCR5^+^PD1^high^ Treg according to the surface marker CXCR5 and programmed cell death protein 1 (PD-1) [9–13]. The function of the two subsets of Tfr remains unclear. Imbalance or disfunction of Tfr subsets may directly or indirectly affect B cells, leading to expansion of overactive B cells which contributes to various immune-related clinical diseases [14, 15]. So far, the role of balance between Tfh and Tfr subsets in SLE is still controversial due to the heterogeneity of the disease, cohort size, and methods of studies [16, 17].

Efficacious treatments, including low-dose interleukin 2 (IL-2), might promote Tfr cell responses, and inhibit Tfh cell development in SLE [18, 19]. However, it is not well understood how these circulating Tfh-like cell subsets, including Tfr and Tfh subsets, are involved in the disease. To date, the balance of these new subsets has not been addressed, neither how these cells respond to SLE treatment. Here, we identified an imbalanced profile of Tfh cell subsets in SLE, including the newly described anti-inflammatory CXCR5^+^PD-1^low^Treg, CXCR5^+^PD-1^high^Treg, and the pro-inflammatory Tfh and Tfh17 and investigated the change of these subtypes by low-dose IL-2 treatment in a randomized, double-blind, placebo-controlled study in SLE.

## Participants and methods

### Participants

This was a post hoc analysis of data from an RCT clinical study (NCT02465580) of low-dose IL-2 in SLE patients. Full details of study designs and inclusion/exclusion criteria for each completed study have previously been published [18]. All active SLE patients who had an inadequate response to standard treatment for ≥3 months were enrolled. Background treatment should be at a stable dose for 12 weeks of immunosuppressant drugs and 4 weeks of glucocorticoids prior to enrollment. Concomitant drugs were kept stable except tapering of glucocorticoids. In addition to standard therapy, IL-2 (1 million IU) or placebo was administered subcutaneously every other day for 2 weeks (seven injections), followed by a 2-week break, as one treatment cycle of 4 weeks. All the patients were treated for the first 12 weeks which included three treatment cycles with IL-2 or placebo and followed-up for another 12 weeks without study medicine. Patients were evaluated at screening, every 2 weeks to week 12, and every 4 weeks thereafter to week 24. Anti-dsDNA antibody levels were measured by enzyme-linked immunosorbent assay (ELISA). The primary end point was attainment of SLE responder index-4 (SRI-4) at week 12 in the trial [20]. Studies were conducted in accordance with the Declaration of Helsinki, the International Conference on Harmonization Guidelines for Good Clinical Practice. Besides, 23 healthy controls (HC) were enrolled during the same time of RCT study. Table 1 summarized baseline characteristics. Written informed consents were obtained from these healthy controls. The experimental protocol followed the guidelines of the Declaration of Helsinki and was approved by the Human Ethics Committee of Peking University People’s Hospital (Beijing, China).

**Table 1:** Baseline characteristics of SLE patients and healthy controls (HC) in this study

Characteristics	SLE (n=60)	HC (n=23)	***P*** value
Age, year, mean ±SD	30.84±9.48	29.83±9.72	0.474
Female/Male	56/4	21/2	>0.99
Duration, months, mean ±SD	65.15±58.65	-	-
Medications		-	-
Prednisone dose, mg/day, median (range)	13.5 (0, 50)	-	-
Hydroxychloroquine	57 (95)	-	-
Cyclophosphamide	4 (6.67)	-	-
Azathioprine	5 (8.33)	-	-
Cyclosporine	5 (8.33)	-	-
Mycophenolate Mofetil	17 (28.33)	-	-
Tacrolimus	2 (3.33)	-	-
Leflunomide	4 (6.67)	-	-
Thalidomide	1 (1.67)	-	-
Methotrexate	1 (1.67)	-	-
Interleukin-2	30 (50)	-	-

For a continuous variable, median (range), or mean±SD. For a categorical variable, count (percentage). *SLE* systemic lupus erythematosus

### Flow cytometric analysis

**Fig. 1:**
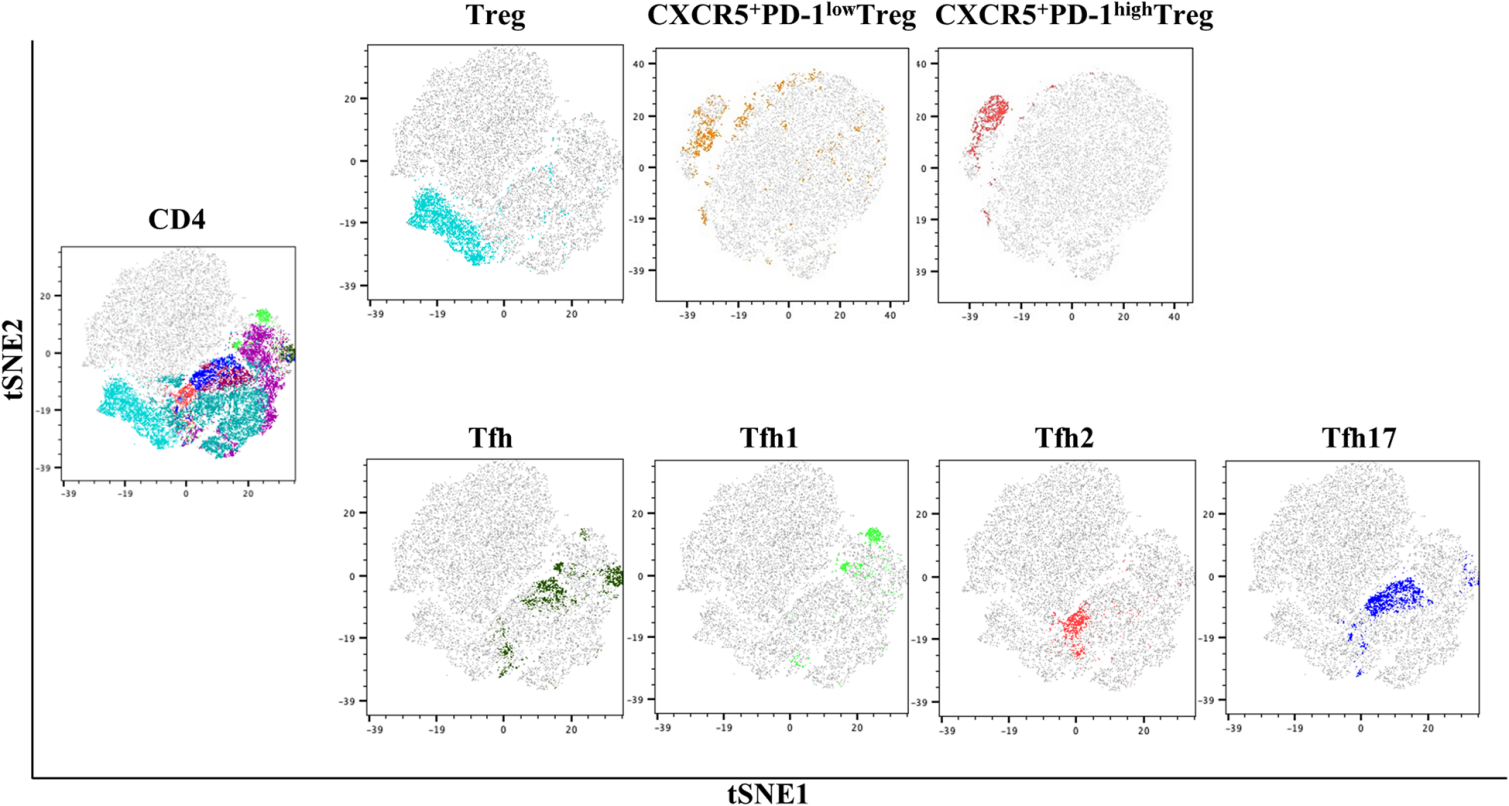
tSNE analysis of CD4 T subsets based on immune cells flow cytometry markers. tSNE, t-stochastic neighbor embedding

Single-cell suspensions from the peripheral blood in SLE patients and HC were analyzed by multicolor flow cytometry (FACSAria II; BD Biosciences, Franklin Lakes, NJ, USA). t-stochastic neighbor embedding (tSNE) analysis of CD4 T subsets based on immune cells flow cytometry markers was shown in Fig. 1. Data were also analyzed using FlowJo v10 software (Tree Star, Ashland, OR, USA) (Figure S1). The absolute number of CD4 T cell subsets was calculated by multiplying proportion of CD4 T cell subsets in lymphocytes by absolute lymphocyte number determined with an automated hematology analyzer. Detailed protocol of trial has been published online [18].

### Cytometric bead array (CBA) analysis of serum cytokines

Serum levels of IL-2, IL-17, and other inflammatory cytokines were determined by human Th1/Th2/Th17 14-plex (QuantoBio, Beijing, China) according to the manufacturer’s instructions.

### Statistical analysis

Data were expressed as the median and range for non-normally distributed data, while mean ± standard deviation (SD.) for normally distributed data. The Student’s unpaired or paired t test was performed to compare two groups for parametric data, and the Mann-Whitney U test or Wilcoxon rank sum test was performed for nonparametric data. Relationships between variables were analyzed by Spearman’s rank test. Statistical analyses were performed using SPSS v.22.0 or R v.3.6.3 software. Two-sided P values < 0.1 were considered statistically significant.

## Results

### Characteristics of SLE patients

Given recent studies showing the imbalances in the effector and regulatory Tfh cell compartment in SLE patients [3, 4], and in light of our finding that low-dose IL-2 treatment significantly influences Tfh subtypes, we recruited a small cohort of healthy controls (HC) (n=23, Table 1) for comparative analysis.

The demographic and clinical manifestations of these patients were shown in Table 1. There was no significant difference between patients and HCs regarding age or gender. 98% of the SLE patients were positive in anti-dsDNA tests, 42% had renal involvement, and 48% had skin manifestations.

### Imbalanced Tfr/Tfh in SLE

As shown in Table 2, regulatory T cells including Treg, CXCR5^+^PD-1^low^Treg, and CXCR5^+^PD-1^high^ Treg cells were reduced in SLE patients than those in HC (P=0.087, P=0.033, and P<0.001, respectively). In contrast, the effector Tfh cells were increased in SLE patients than in HC (P=0.081). Besides, Tfr subsets:Tfh subset ratios in SLE were dramatically decreased, including CXCR5^+^PD-1^low^ Treg/Tfh (P=0.043), CXCR5^+^PD-1^high^ Treg/Tfh (P<0.001), CXCR5^+^PD-1^low^ Treg/Tfh17 (P=0.052), and CXCR5^+^PD-1^high^ Treg/Tfh17 (P<0.001).

**Table 2:** Difference of CD4 T subsets between HC and SLE, and between active and remission group

Variables	HC (n=23)	SLE		***P***, HC VS. Active	***P***, remission VS. active
		Active (n=60) (Before therapy)	Remission (n=59) (After therapy)		
**Proportion (percentage in lymphocyte, %)**					
**Treg**	1.22 (1, 1.47)	0.95 (0.65, 1.53)	1.6 (0.97, 2.49)	0.087	0.001
**CXCR5**^**+**^**PD-1**^**low**^**Treg**	0.09 (0.04, 0.14)	0.06 (0.03, 0.1)	0.13 (0.06, 0.25)	0.033	<0.001
**CXCR5**^**+**^**PD-1**^**high**^**Treg**	0.01 (0.007, 0.013)	0.003 (0.002, 0.008)	0.02 (0.009, 0.052)	<0.001	<0.001
**Tfh**	0.28 (0.17, 0.43)	0.39 (0.24, 0.56)	0.19 (0.09, 0.37)	0.081	0.002
**Tfh1**	0.61 (0.38, 0.99)	0.2 (0.08, 0.36)	0.17 (0.09, 0.28)	<0.001	0.42
**Tfh2**	1.06 (0.58, 1.5)	0.42 (0.21, 0.6)	0.36 (0.19, 0.68)	<0.001	0.367
**Tfh17**	0.45 (0.15, 0.65)	0.47 (0.27, 0.87)	0.44 (0.2, 0.83)	0.345	0.851
**Absolute number (cells/μl)**					
**Treg**	25.51 (19.98, 30.78)	11.94 (6, 20.81)	20.51 (9.17, 29.06)	0.035	0.014
**CXCR5**^**+**^**PD-1**^**low**^**Treg**	2.98 (1.71, 3.1)	0.67 (0.24, 1.21)	1.33 (0.8, 3.54)	0.037	0.005
**CXCR5**^**+**^**PD-1**^**high**^**Treg**	0.39 (0.28, 0.43)	0.04 (0.02, 0.11)	0.24 (0.11, 0.72)	0.006	<0.001
**Tfh**	7.29 (4.34, 8.92)	4.6 (2.58, 10.28)	2.84 (0.81, 6.04)	0.625	0.008
**Tfh1**	14.26 (12.37, 18.22)	2.11 (0.95, 5.47)	1.92 (0.68, 4.28)	0.002	0.427
**Tfh2**	19.31 (14.66, 24.22)	4.43 (2.11, 11.2)	4.04 (1.73, 11.88)	0.002	0.751
**Tfh17**	8.82 (7.25, 12.22)	5.39 (2.33, 14.38)	4.67 (2.47, 10.36)	0.642	0.664
**Ratios**					
**Treg/Tfh**	3.69 (3.04, 8.49)	2.49 (1.89, 4.5)	8.93 (4.04, 18.29)	0.002	<0.001
**CXCR5**^**+**^**PD-1**^**low**^**Treg/Tfh**	0.3 (0.21, 0.57)	0.16 (0.08, 0.31)	0.61 (0.4, 1.32)	0.002	<0.001
**CXCR5**^**+**^**PD-1**^**high**^**Treg/Tfh**	0.06 (0.04, 0.08)	0.01 (0, 0.02)	0.1 (0.06, 0.2)	<0.001	<0.001
**Treg/Tfh17**	3.87 (2.27, 5.71)	2.29 (1.23, 4.21)	3.89 (2.01, 6.62)	0.047	<0.001
**CXCR5**^**+**^**PD-1**^**low**^**Treg/Tfh17**	0.25 (0.17, 0.34)	0.11 (0.05, 0.22)	0.28 (0.18, 0.52)	0.002	<0.001
**CXCR5**^**+**^**PD-1**^**high**^**Treg/Tfh17**	0.04 (0.03, 0.06)	0.01 (0, 0.02)	0.04 (0.02, 0.08)	<0.001	<0.001

*SLE* systemic lupus erythematosus, *HC* healthy controls. Data are median (IQR)s

### High Tfh associated with decreased serum IL-2

**Fig. 2:**
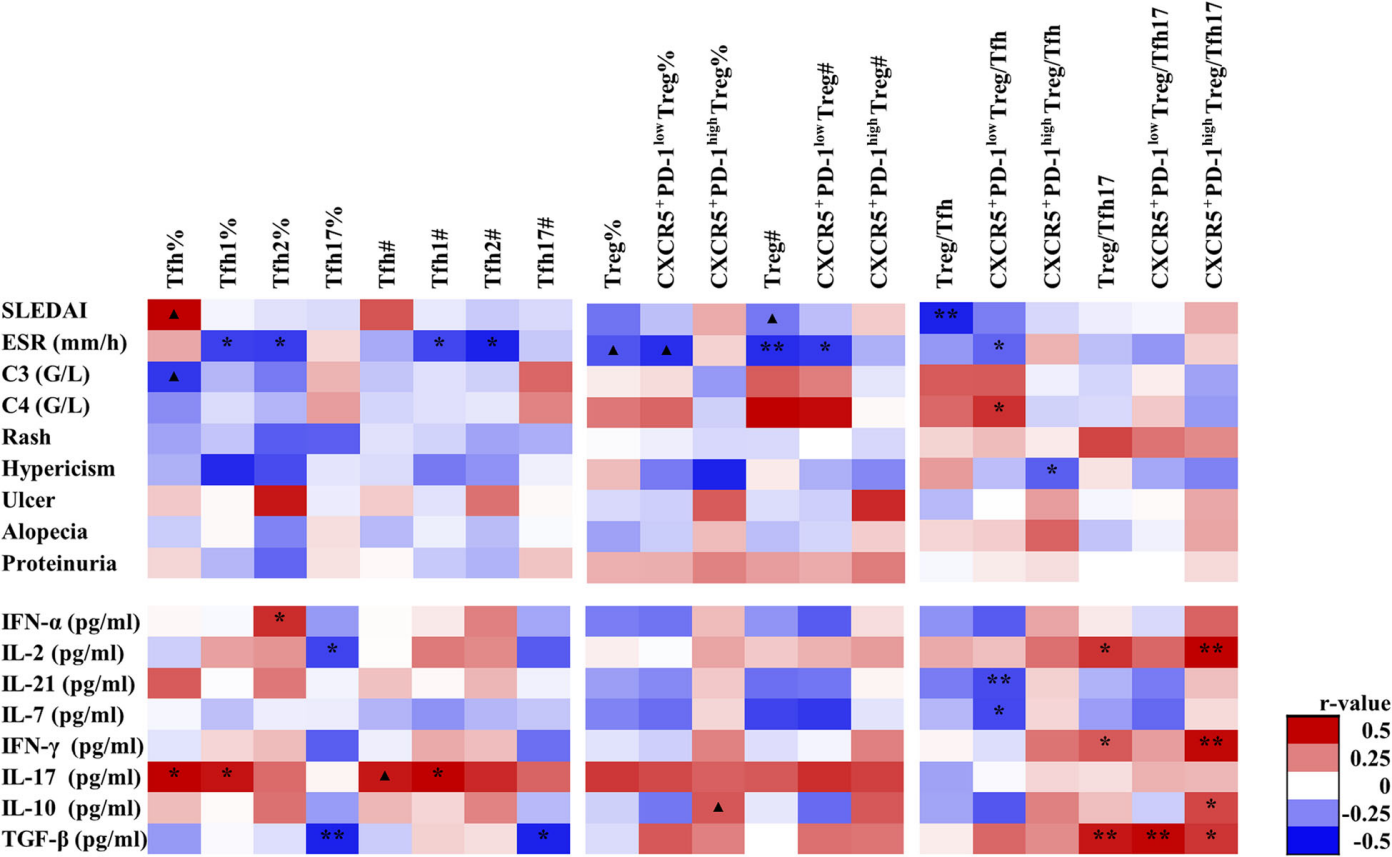
The correlations between CD4 T cell subsets and clinical characters in SLE. SLEDAI, SLE Disease Activity Index. ESR, erythrocyte sedimentation rate. C3, complement 3. C4, complement 4. IFN-α, interferon-α. IL-2, Interleukin-2. IL-21, interleukin-21. IL-7, interleukin-7. IFN-γ, interferon-γ. IL-17, interleukin-17. IL-10, interleukin-10. TGF-β, tumor necrosis factor-β. The scale color of the filled squares indicates the strength of the correlation (r) and whether it is negative (blue) or positive (red). **, P<0.01. *, P<0.05. ▲, P<0.1. #, absolute number, cells/μl. %, proportion of cells in lymphocyte

**Fig. 3:**
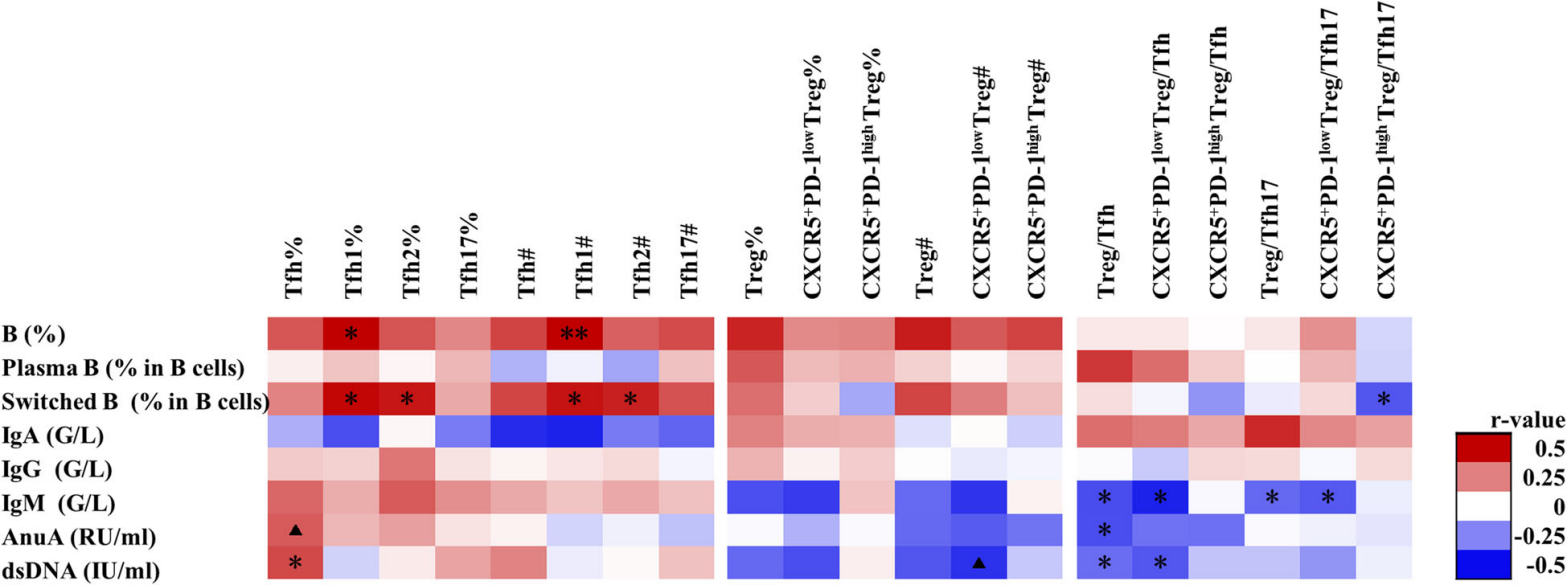
The correlations between CD4 T cell subsets and B cells and immunoglobulins in SLE. B, B cell. Plasma B, plasma B cell. Switched B, switched memory B cells. IgA, immunoglobulin A. IgG, immunoglobulin G. IgM, immunoglobulin M. AnuA, anti-nucleosome antibodies. dsDNA, anti-double stranded DNA antibodies. The scale color of the filled squares indicates the strength of the correlation (r) and whether it is negative (blue) or positive (red). *, P<0.05. **, P<0.01. ▲, P<0.1. #, absolute number, cells/μl. %, proportion of cells in lymphocyte

As show in Fig. 2, an expanded inflammatory Tfh cell compartment was correlated with higher serum IL-17 levels (r=0.328, P=0.021) and increased frequency of Tfh17 correlated with lower serological IL-2 (r=−0.295, P=0.04) (Fig. 2). There was a reduction of CXCR5/PD-1 double positive subset (CXCR5^+^PD1^high^ Treg), which was related with IL-10 elevation (r=0.243, P=0.093, Fig. 3).

### Imbalanced Tfh and Tfr cell association with disease activity in SLE

Treg cells were decreased and associated with elevated ESR (r=−0.382, P<0.01) and Safety of Estrogens in Lupus Erythematosus National Assessment version of the SLE Disease Activity Index (SLEDAI) (r=−0.245, P=0.089, Fig. 2). At the same time, the decrease of CXCR5^+^PD-1^low^Treg was associated with increased ESR and anti-dsDNA antibody production (Figs. 2 and 3). In contrast, an increased inflammatory Tfh cell compartment was found and was correlated with elevated SLEDAI, titer of anti-AnuA, anti-dsDNA antibodies, serum IL-17, and decreased C3 (Figs. 2 and 3).

Upon further analysis of the correlation between regulatory and effector subsets, we found decreased Treg/Tfh ratio in severe patients with higher SLEDAI score, higher titers of anti-AnuA, and anti-dsDNA antibodies (Fig. 3). In addition, there was a reduced CXCR5^+^PD1^low^Treg/Tfh in this group of severe patient.

Figure 3 showed that the frequency of Tfh1 and Tfh2 was positively correlated with the number of total B cells and switched memory B (CD19^+^IgD^-^CD27^+^) cells. CXCR5^+^PD-1^low^Treg/Tfh17 was negatively correlated with switched memory B cells and plasma B cells (r=−0.341, P=0.027, Fig. 3). And decreased CXCR5^+^PD-1^low^ Treg/Tfh and CXCR5^+^PD-1^low^Treg/Tfh17 were both associated with increased serum level of IgM.

### Low-dose IL-2 therapy increased Tfr/Tfh ratio in SLE patients

**Fig. 4:**
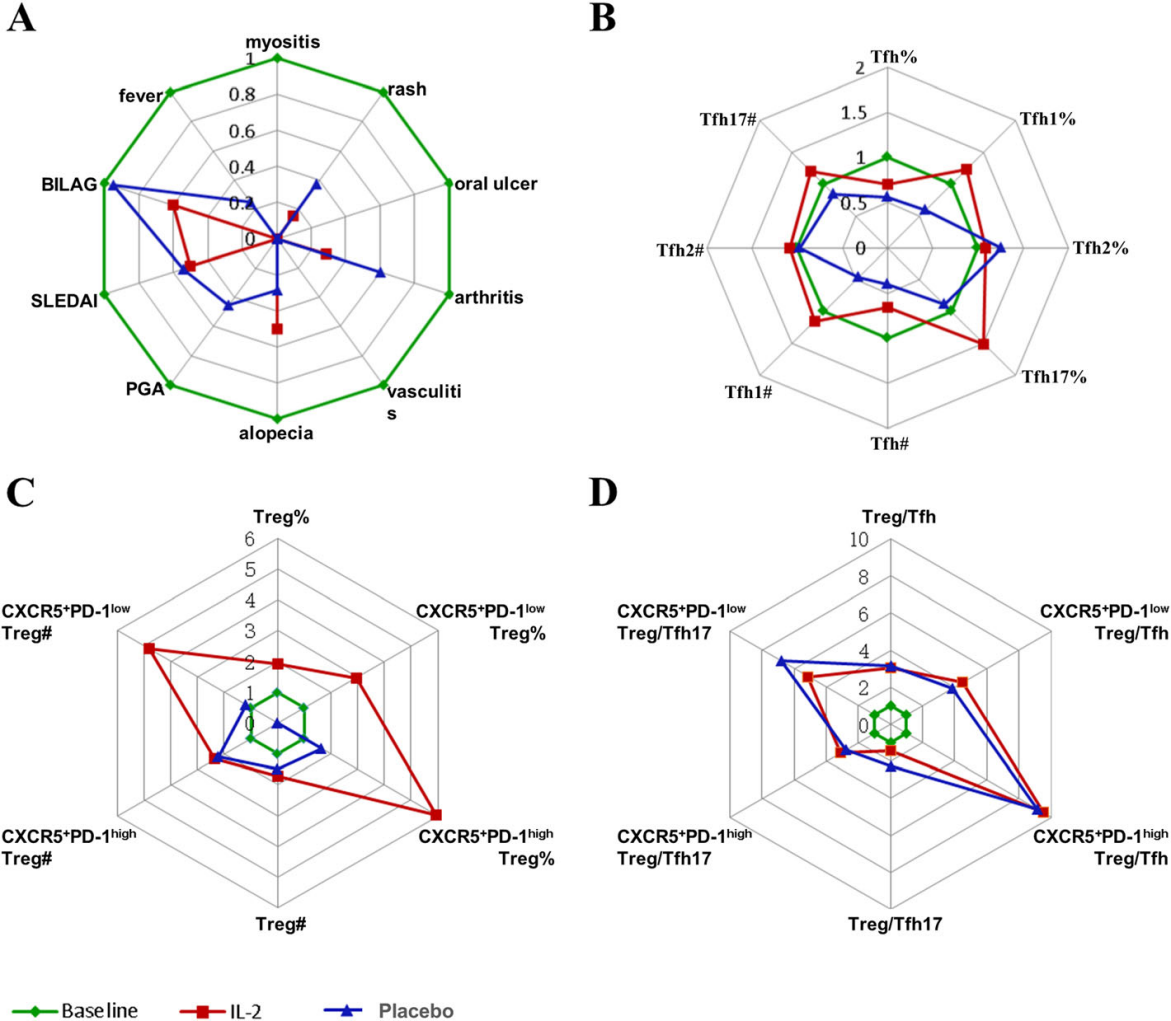
The response of clinical and immune cells to low-dose IL-2 (n=30) and placebo (n=30) therapy in SLE. **A** Relative change of disease activity value and patient number. **B** Relative change of ratios at baseline and week 12. **C** Relative change of Treg subsets at baseline and week 12. **D** Relative change of Tfh subsets at baseline and week 12. BILAG, British Isles Lupus Assessment Group. SLEDAI, Safety of Estrogens in Lupus Erythematosus National Assessment version of the SLE Disease Activity Index. SRI-4, SLE Responder Index-4. PGA, physician’s global assessment. All data here were normalized to baseline values to show relative changes. In detail, all baseline data and data after therapy were divided by the value of baseline. #, absolute number, cells/μl. %, proportion of cells in lymphocyte

In this RCT of low-dose IL-2 therapy in SLE, low-dose IL-2 significantly increased Tregs [18]. With effective treatment, British Isles Lupus Assessment Group (BILAG), SLEDAI, SLE Responder Index-4 (SRI-4), physician’s global assessment (PGA), myositis, fever, alopecia, vasculitis, arthritis, oral ulcer, and rash were all improved at week 12 (Fig. 4) [18]. The levels of serum C3 recovered in 56.25% (9/16) patients. After 3 cycles of low-dose IL-2 therapy, the frequency of CXCR5^+^PD-1^low^Treg cells and CXCR5^+^PD-1^high^ Treg cells in lymphocyte was significantly increased at week 12 compared to the placebo control (0.06 (0.03, 0.1) vs. 0.13 (0.06, 0.25), *P*<0.001 and 0.003 (0.002, 0.008) vs. 0.02 (0.009, 0.052), *P*<0.001, respectively) (Table 2, Fig. 4). Similarly, the absolute number of these Treg cells (CXCR5^+^PD-1^high^ Treg and CXCR5^+^PD-1^low^Treg) were significantly increased after the treatment of low-dose IL-2 (0.67 (0.24, 1.21) vs. 1.33 (0.8, 3.54), P=0.005 and 0.04 (0.02, 0.11) vs. 0.24 (0.11, 0.72), P<0.001, respectively) (Table 2, Fig. 4). Besides, compared to baseline, Tfr subsets:Tfh subset ratios in SLE were dramatically increased, including CXCR5^+^PD-1^low^ Treg/Tfh (P<0.001), CXCR5^+^PD-1^high^ Treg/Tfh (p<0.001), CXCR5^+^PD-1^low^ Treg/Tfh17 (P<0.001), and CXCR5^+^PD-1^high^ Treg/Tfh17 (P<0.001).

## Discussion

Increasing evidence indicates that Tfh cells are important in the pathogenesis of SLE. Tfh cells are recognized as a distinct T cell subset, which provides help for GC formation, B cell affinity maturation, and immunoglobulin class switching, as an indispensable part of adaptive immunity. Our previous work showed that patients with SLE have an increased number of peripheral Tfh cells, which positively correlates with autoantibody titers (anti-dsDNA antibodies) and disease activity, as measured by the SLEDAI. Others have reported that the aberrant expression of Tfh cells is a common feature in mouse models of SLE, suggesting its contribution in the development of autoimmune diseases [4, 5].

Besides Tfh cells, a subset of Treg cells, named Tfr cells, have been identified. These cells share common characteristics with Tfh and conventional Treg cells and can inhibit GC responses, regulating the number of Tfh and GC B cells [6–8]. Therefore, it is generally believed that Tfr cells constrain the B cell “help” provided by Tfh cells to maintain immune homeostasis. An aberrant or disordered Tfh/Tfr balance may result in the break of tolerance, excessive B cell proliferation, antibody production, and the development of autoimmune diseases. A recent study showed the importance of the Tfr/Tfh balance in autoimmune responses in BXD2 mice, which display spontaneous autoreactive GC formation [21]. In addition, intravenous immunoglobulin administration to mice with collagen-induced arthritis augments the number of Tfr cells and represses the subsequent maturation of GC B cells [22], which also supports the idea of a critical role for Tfr cells in autoimmune diseases.

There was no difference in absolute number of Tfh17 subsets between SLE and healthy controls. The proportion of Tfh17 in CD4 T cells was significantly higher in SLE than in HC, although there was no significant difference in Tfh17 proportion in lymphocytes. Therefore, in this study, the focus is on the Tfr/Tfh balance in the SLE.

In B cell co-culture, the Tfh2 and Tfh17 cells exceeded the Tfh1 cells to support antibody production [23]. A general trend of increasing proportions of Tfh2 and Tfh17 cells and decreasing proportions of Tfh1 cells was observed in SLE [24]. The numbers of Tfh1 and Tfh2 cells are relatively lower in SLE than in HC in our study. It is possible that Tfh2 cells do not play an important pathogenic role in this cohort of patients.

There was no significant difference between CXCR5^+^PD-1^low^ Treg and CXCR5^+^PD-1^high^ Treg in our study. In a study, it was found that the majority of Tfr cells in the lymph nodes express low levels of PD-1 and reside at the border between the T cell zone and B cell follicle, with very few found in the germinal centers (GCs) [9]. Although PD-1^+^ Tfr cells expressed higher levels of CD38, CTLA-4, and GARP than PD-1^-^ Tfr cells, both potently suppressed antibody production in vitro. These results demonstrate the phenotypic diversity of human Tfr cells [9].

In our study, we found a deficiency of Tfr cell subsets, including CXCR5^+^PD-1^low^Treg and CXCR5^+^PD-1^high^ Treg, and increased Tfh cells in the peripheral blood of SLE patients. The shifted balance between circulating CXCR5^+^PD-1^low^ Treg and Tfh cells correlated not only with reduced serum IL-2, IL-10, and increased IL-21 levels in patients but also with clinical SLE parameters, e.g., ESR, anti-dsDNA antibodies, and disease activity (SLEDAI Scores). These findings are consistent with previous studies in vitro, in which Tfh and CXCR5^+^PD-1^low^ Treg or CXCR5^+^PD1^high^ Treg cells can antagonize B cell function, production of high-affinity antibodies, and the memory B cell differentiation [22]. CXCR5^+^PD-1^low^ Treg cells play an important immunosuppressive function by curbing self-reactive auto-antibodies development within the GC during an inflammatory immune response [5]. Therefore, deregulation of the Tfr and Tfh cell compartments is associated with disease severity, B cell frequency, and antibody production in SLE.

There have been several relatively successful attempts to reduce the severity of SLE in humans via blockade of Tfh-cell differentiation and activity. Studies using monoclonal antibodies against ICOSL inhibited the development of Tfh and GC B cells resulting in decreased anti-dsDNA antibodies and improved kidney function in both human and mouse [23]. For years, SLE therapy has relied on broad spectrum immunosuppressants; however, a growing body of work shows that a targeted increase of regulatory T cells may be a more attractive therapy [18, 19, 24, 25].

IL-2 is essential for the development and maintenance of Treg cells, which prevent the development of autoimmune disease. Low-dose IL-2 can promote Tregs by activating the transcription factor STAT5, which binds to the Foxp3 locus and promotes Foxp3 expression without activation of effector T cells. More recently, IL-2 has been shown to be essential for the inhibition of Tfh cell development. Thus, in this study, we asked if low-dose IL-2 therapy might also elevate the Tfr/Tfh ratio, exploring a novel concept for rational therapeutic design.

Our previous studies had proven a deficient Treg cell compartment and decreased IL-2 levels in circulation of SLE, and the efficacy of low-dose IL-2 treatment. But there was no study addressing the impact of low-dose IL-2 on Tfr:Tfh balance. After effective therapy, especially low-dose IL-2 therapy, the imbalanced Tfr and Tfh subsets were reversed accompanying improvement of disease activity. Furthermore, Tfr subsets were all increased regardless of output measurement; proportion and absolute number. Although we did not see a significant change in Th17 frequency, the ratios of CXCR5^+^PD-1^low^ Treg/Tfh17 and CXCR5^+^PD-1^high^ Treg/Tfh17 were significantly decreased compared to those in healthy controls. Besides, we did not see any obvious difference between CXCR5^+^PD-1^low^ Treg and CXCR5^+^PD-1^high^ Treg, perhaps reflecting a functional overlap of these two subsets.

The strength of our study was to systematically analyze the change in Tfr and Tfh subsets in SLE and its correlation with relevant clinical parameters. The main limitation of this article was a relatively small number of patients with a very heterogeneous disease, which may give rise to a caution about over-interpretation of the data. Another limitation is that we definite Treg cell as CD4^+^CD25^high^CD127^low^, but not CD4^+^CD25^high^CD127^low^ Foxp3^+^, both stain methods should be applied in the future studies.

## Conclusions

In summary, our findings indicate that imbalance of Tfh and Tfr is important for SLE severity, and low-dose IL-2 ameliorates lupus autoimmunity favoring Tfr cell expansion. Our study added to these findings by demonstrating that low-dose IL-2 therapy selectively activates and expands Tfr cells, while demonstrating clinical efficacy in SLE. Further studies are needed to better understand how to explore Tfh cell or Tfr cell signatures to stratify patients, and guide the design of novel treatment regiments for SLE in future clinical trials.

## Supplementary Information

**Additional file 1: Figure S1.** Representative gating.

**Additional file 2: Table S1.** Difference of CD4 T subsets between SLE patients with and without renal disease.

## Data Availability

Data are available upon reasonable request.

## Abbreviations

Tfr: T follicular regulatory
Tfh: T follicular
IL-2: Interleukin-2
SLE: Systemic lupus erythematosus
tSNE: t-Stochastic neighbor embedding
HC: Healthy controls
IL-17: Interleukin-17
SLEDAI: SLE Disease Activity Index
IgM: Immunoglobulin M
Treg: Regulatory T
GC: Germinal center
SD: Standard deviation
SRI-4: SLE responder index-4
Anti-AnuA: Anti-nucleosome antibody
Anti-dsDNA: Anti-double-stranded DNA antibody
C3: Complement 3
BILAG: British Isles Lupus Assessment Group
PGA: Physician’s global assessment
ESR: Erythrocyte sedimentation rate
PD-1: Programmed cell death protein 1
