# Supplementary figures and images for: Therapeutic potential of targeting Tfr/Tfh cell balance by low-dose-IL-2 in active SLE: a post hoc analysis from a double-blind RCT study
Source: Arthritis Res Ther. 2021 Jun 11;23:167. doi: 10.1186/s13075-021-02535-6 (PMC8194162; doi:10.1186/s13075-021-02535-6)

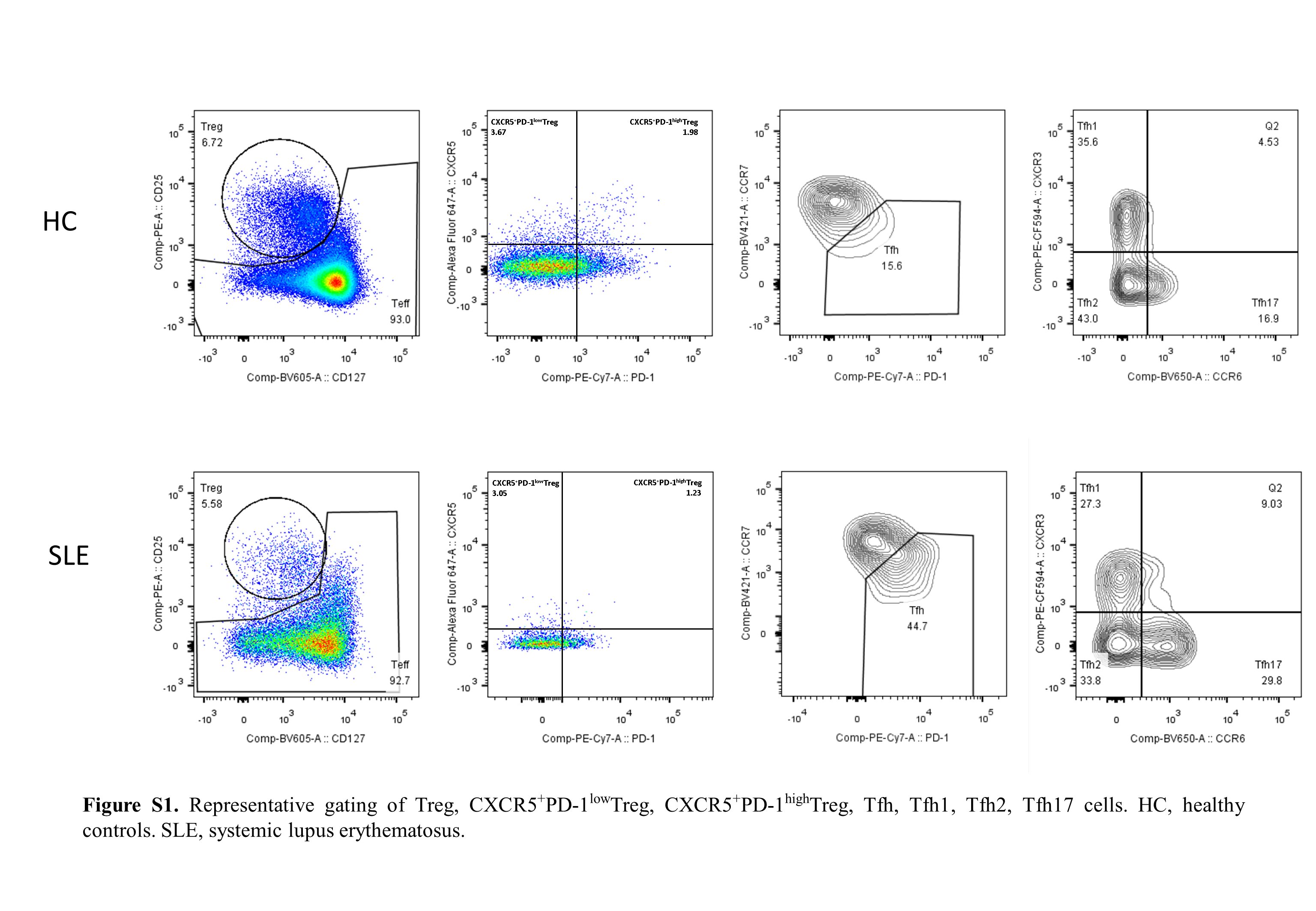

Supplement: Supplementary file 1 — Additional file 1: Figure S1. Representative gating. [file 13075_2021_2535_MOESM1_ESM.tif]
