# Supplementary material for: Therapeutic potential of targeting Tfr/Tfh cell balance by low-dose-IL-2 in active SLE: a post hoc analysis from a double-blind RCT study
Source: Arthritis Res Ther. 2021 Jun 11;23:167. doi: 10.1186/s13075-021-02535-6 (PMC8194162; doi:10.1186/s13075-021-02535-6)
Supplement: Supplementary file 2 — Additional file 2: Table S1. Difference of CD4 T subsets between SLE patients with and without renal disease. [file 13075_2021_2535_MOESM2_ESM.docx]

| **Table S1. Difference of CD4 T subsets between SLE patients with and without renal disease.** | | | | | |
| --- | --- | --- | --- | --- | --- |
| **Variables** | **Non-renal disease (n=35)** | **Renal disease (n=25)** | | ***P* Value** | |
| **Proportion (percentage in lymphocyte, %)** | | | |  | |
| **Treg** | 0.88 (0.54, 1.7) | | 1.06 (0.68, 1.42) | 0.678 | |
| **CXCR5^+^PD-1^low^Treg** | 0.07 (0.02, 0.11) | | 0.06 (0.03, 0.08) | 0.421 | |
| **CXCR5^+^PD-1^high^Treg** | 0.003 (0.002, 0.008) | | 0.004 (0.002, 0.008) | 0.605 | |
| **Tfh** | 0.38 (0.19, 0.58) | | 0.41 (0.27, 0.57) | 0.652 | |
| **Tfh1** | 0.21 (0.07, 0.4) | | 0.16 (0.09, 0.31) | 0.312 | |
| **Tfh2** | 0.45 (0.21, 0.7) | | 0.4 (0.21, 0.47) | 0.287 | |
| **Tfh17** | 0.46 (0.27, 0.98) | | 0.44 (0.17, 0.81) | 0.448 | |
| **Absolute number (cells/L)** |  | | | | |
| **Treg** | 11.94 (4.06, 25.9) | | 11.92 (6.04, 19.88) | | 0.97 |
| **CXCR5^+^PD-1^low^Treg** | 0.78 (0.24, 2.2) | | 0.66 (0.37, 1.14) | | 0.544 |
| **CXCR5^+^PD-1^high^Treg** | 0.04 (0.02, 0.14) | | 0.04 (0.02, 0.12) | | 0.691 |
| **Tfh** | 4.75 (2.12, 11.95) | | 4.29 (2.49, 10.16) | | 0.69 |
| **Tfh1** | 2.18 (0.98, 6.26) | | 1.76 (0.85, 4.88) | | 0.361 |
| **Tfh2** | 5.24 (2.47, 11.33) | | 4.17 (1.77, 8.84) | | 0.482 |
| **Tfh17** | 6.09 (2.67, 17.04) | | 4.81 (1.83, 11.7) | | 0.436 |
| **Ratios** |  | |  | |  |
| **Treg/Tfh** | 2.49 (1.68, 4.68) | | 2.49 (1.95, 4.6) | | 0.814 |
| **CXCR5^+^PD-1^low^Treg/Tfh** | 0.17 (0.08, 0.31) | | 0.15 (0.08, 0.32) | | 0.984 |
| **CXCR5^+^PD-1^high^Treg/Tfh** | 0.008 (0.003, 0.017) | | 0.009 (0.004, 0.021) | | 0.882 |
| **Treg/Tfh17** | 2.06 (1.18, 3.92) | | 2.66 (1.38, 4.59) | | 0.376 |
| **CXCR5^+^PD-1^low^Treg/Tfh17** | 0.12 (0.06, 0.24) | | 0.11 (0.05, 0.23) | | 0.81 |
| **CXCR5^+^PD-1^high^Treg/Tfh17** | 0.007 (0.003, 0.013) | | 0.008 (0.002, 0.022) | | 0.63 |

| **Table S2. Difference of Tfh and Tfr subsets between HC and SLE, and between before and after therapy.** | | | | | |
| --- | --- | --- | --- | --- | --- |
| Variables (% in CD4 T) | HC | SLE | | *P*, HC vs. Active | *P*, Remission vs. Active |
|  |  | Active | Remission |  |  |
| Treg | 5.19 (4.32, 6.79) | 5.62 (4.25, 6.66) | 11 (7.62, 15.82) | 0.595 | <0.001 |
| CXCR5^+^PD-1^low^ Treg | 0.39 (0.26, 0.62) | 0.37 (0.2, 0.75) | 0.89 (0.35, 1.71) | 0.984 | <0.001 |
| CXCR5^+^PD-1^high^ Treg | 0.04 (0.03, 0.07) | 0.02 (0.01, 0.05) | 0.1 (0.06, 0.32) | 0.002 | <0.001 |
| Tfh | 1.29 (0.8, 1.74) | 2.33 (1.43, 2.93) | 1.11 (0.61, 2.11) | <0.001 | 0.015 |
| Tfh1 | 2.57 (1.73, 3.57) | 1.17 (0.78, 2.12) | 13.65 (8.21, 20.83) | <0.001 | 0.361 |
| Tfh2 | 4.19 (3.25, 5.09) | 2.45 (1.95, 3.17) | 32.8 (21.55, 46.45) | <0.001 | 0.580 |
| Tfh17 | 1.75 (1.22, 3.87) | 3.09 (1.87, 5.27) | 39.1 (25.95, 53.48) | 0.019 | 0.630 |
